# Paralytic shellfish poisonings resulting from an algal bloom in Nicaragua

**DOI:** 10.1186/s13104-015-1012-4

**Published:** 2015-03-10

**Authors:** Luis Callejas, Ana Cristian Melendez Darce, Juan Jose Amador, Laura Conklin, Nicholas Gaffga, Helen Schurz Rogers, Stacey DeGrasse, Sherwood Hall, Marie Earley, Joanne Mei, Carol Rubin, Sylvain Aldighieri, Lorraine C Backer, Eduardo Azziz-Baumgartner

**Affiliations:** Field Epidemiology Training Program, Managua, Nicaragua USA; Nicaragua Ministry of Health, Managua, Nicaragua USA; U.S. Centers for Disease Control and Prevention, 1600 Clifton Rd, NE, MS A32, Atlanta, GA 30333 USA; U.S. Food and Drug Administration, College Park, Maryland USA; Pan American Health Organization, Managua, NI USA

**Keywords:** Paralytic shellfish poisoning, Saxitoxin, Nicaragua, Algal bloom, *Anadara tuberculosa*, *Pyrodinium bahamense*

## Abstract

**Background:**

During an October 2005 algal bloom (i.e., a rapid increase or accumulation in the population of algae) off the coast of Nicaragua, 45 people developed symptoms of paralytic shellfish poisoning (PSP) and one person died. PSP in humans is caused by ingestion of saxitoxin, which is a neurotoxin often associated with shellfish contaminated by algal blooms.

To explore the relationship between the algal bloom and human illnesses, we performed a case-control study of residents living in a coastal island. We administered a standardized clinical questionnaire, sampled locally harvested seafood and algae, and obtained urine samples for saxitoxin testing from symptomatic and asymptomatic persons. PSP case-patients were defined as island residents who developed at least one neurological symptom during the November 4–16 intoxication period. Seafood and algal samples were analyzed for saxitoxins using the receptor-binding assay and high-performance liquid chromatography. Two urine samples were analyzed for saxitoxins using a newly developed immunoassay.

**Findings:**

Three shellfish and two algal samples tested positive for saxitoxins. Ten (9%) of 107 participants developed neurological symptoms during the specified time period and five required hospitalization. While 6 (67%) of 9 possible case-patients and 21 (21%) of 98 controls had eaten fish (p=0.008), all case-patients and 17 (17%) of controls had eaten clams (P<0.0001). The saxitoxin concentration in the urine of a hospitalized case-patient was 21 ng saxitoxin/g creatinine compared to 0.16 ng saxitoxin/g creatinine in the single control patient’s urine.

**Conclusions:**

These findings suggest that a bloom of saxitoxin-producing algae resulted in saxitoxin accumulation in local clams and was responsible for the PSP intoxication.

## Background

The paralytic shellfish poisons, collectively referred to as saxitoxins or paralytic shellfish toxins, are neurotoxins produced by dinoflagellate species from the *Alexandrium*, *Pyrodinium*, and *Gymnodinium* genera. The biological activity of these toxins is based on selective blockage of the voltage-gated sodium channel of many excitable membranes [1]. Humans, other mammals, birds, and fish can be affected by paralytic shellfish poisoning (PSP) toxins.

Paralytic shellfish toxins accumulate in bivalves (e.g., clams, mussels) and certain gastropods that ingest and filter seawater containing these microalgae [2-5]. Humans may become ill with PSP when they ingest phytoplankton-feeding shellfish in which saxitoxins have accumulated [6].

In humans, PSP symptoms usually occur within 30 minutes to about 3 hours after eating contaminated seafood, and symptom severity depends upon the amount of toxin ingested and absorbed by the body. The initial symptoms of PSP are paresthesia and numbness around the lips and mouth [1]. In addition to neurological symptoms, victims may experience nausea and vomiting, dizziness, incoherent speech, light-headedness, and respiratory distress. In severe poisonings, paralysis can affect core muscles such as the thoracic diaphragm and death can result from respiratory failure. The human case fatality proportion from PSP varies considerably with local health-care practitioner awareness of the intoxication and its treatment and the local medical system’s capacity to assist one or more poisoning victims needing respiratory support. For example, in a series of intoxications in Europe and North America, no deaths occurred among more than 200 cases. In similar outbreaks in Southeast Asia and Latin America, where clinicians may be unfamiliar with such intoxication, the case fatality proportion ranged between 2-14% [1].

**Figure 1:**
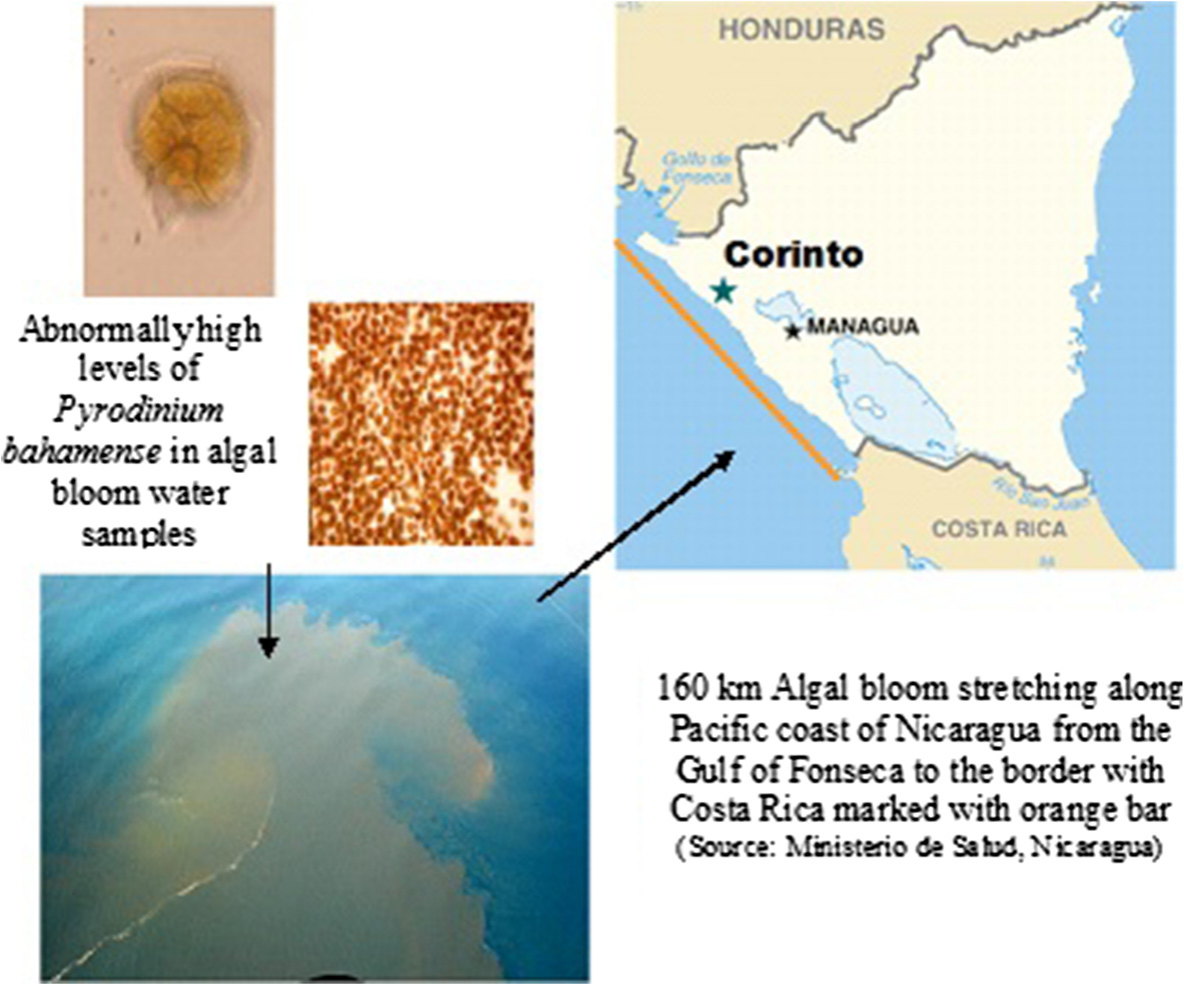
***Pyrodinium bahamense*** **in algal bloom water samples along the coast of Nicaragua, October-November, 2005.**

In mid-October 2005, a local fisherman working near Corinto, a small town on the Pacific coast of Nicaragua, noticed a fluorescent greenish hue to the ocean water that significantly impaired underwater visibility (Figure 1). Within weeks, persons who experienced neurologic symptoms after eating seafood appeared in emergency rooms in and around Corinto. Seawater samples from the area contained 17.5 × 10^6^ dinoflagellates per liter, almost twice the density needed to consider an algal bloom harmful [7]. Ninety-nine percent of these organisms were *P. bahamanse*, which is known to produce the saxitoxins that cause PSP.

**Figure 2:**
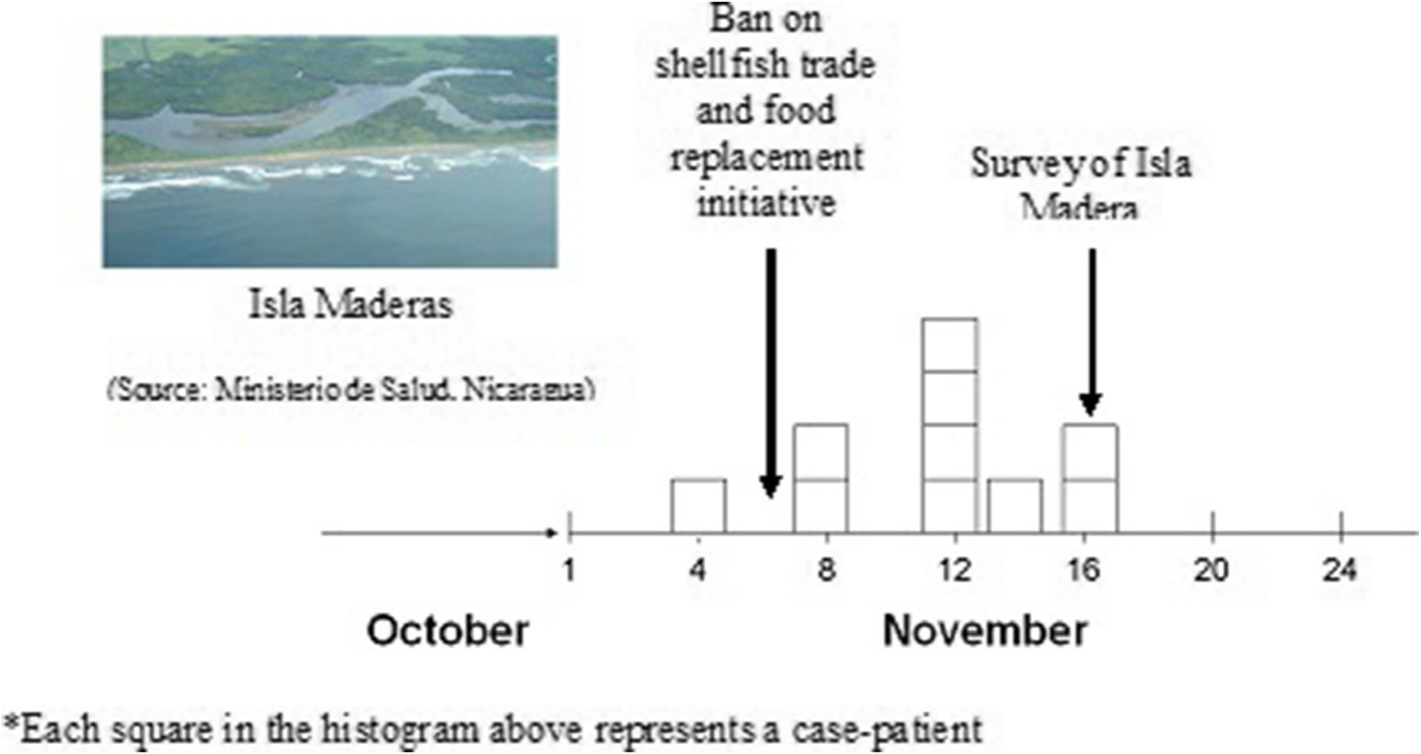
**Timeline of illness onset among participants during the paralytic shellfish poisoning intoxication, ban on shellfish trade, and Isla Maderas survey, Nicaragua, 2005.**

**Figure 3:**
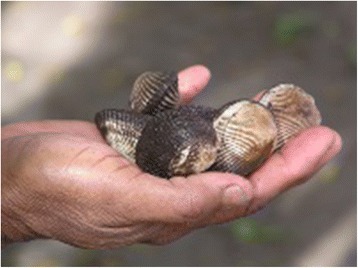
***Anadara tuberculosa*** **(concha negra) implicated in Isla Madera paralytic shellfish poisoning in Nicaragua, November, 2005.**

During November 4–16, 45 possible PSP cases were reported from El Playón, Corinto, El Realejo, Chichigalpa, Chinandega, and El Viejo, Nicaragua, including one case with severe respiratory distress and one death. The most affected area was a small island off the coast of Corinto called Maderas Negras Island (Figure 2) where 10% of the population became ill after eating shellfish harvested in local bays (Figure 3). Samples of shellfish obtained from nearby areas were analyzed in Managua, Nicaragua, for the presence of neurotoxins using the mouse bioassay [8] and tested strongly positive for a toxicant. All 15 mice died after an intraperitoneal injection of 1 ml of an acid extraction from the implicated shellfish samples, with survival time ranging from 1–15 minutes.

On November 6, two days after the outbreak onset, the Nicaraguan Ministry of Health released a public health advisory banning shellfish harvesting and consumption. Businesses selling shellfish would be fined between 500 and 50,000 Nicaraguan Cordobas ($21–$28,855USD). To help those who depended on shellfish for nutrition comply with the ban, the government distributed food relief consisting of sugar, rice, oil, and beans to approximately 800 local families. In addition to local economic consequences, the ban also had important national economic implications because Nicaragua exported shellfish to neighboring countries such as El Salvador.

To verify that shellfish were implicated with the intoxications and determine if other types of seafood could also be contaminated, the Government of Nicaragua partnered with the U.S. Centers for Disease Control and Prevention (CDC) and the U.S. Food and Drug Administration (FDA) to conduct an epidemiologic investigation. Outbreak investigators hypothesized that island inhabitants were becoming ill by eating a specific type or types of shellfish. In this article, we present the findings of the investigation, which aimed to verify that PSP was responsible for the outbreak in Nicaragua, to determine the source of PSP, and to identify modifiable risk factors that once identified could help prevent future illnesses.

## Methods

### Study site

We first conducted a cross-sectional assessment of Maderas Negras Island, located off the coast of Corinto, Nicaragua (Figure 1). This remote island posed a unique opportunity to study a disease outbreak because the island is isolated and has a stable population of residents. Travel to and from the island was limited to high tide, when the island was accessible by boat. We surveyed all households in Maderas Negras Island. Residents typically ate one large meal a day, often relying on locally harvested seafood for their nutrition.

### Survey tool

Our teams traveled to the island during November 16–17 to administer a door-to-door clinical history and food intake questionnaire to ascertain case-status and exposure history among all residents aged ≥1 year. If participants were aged 1–7 years, we asked parents to help children answer study questions. We asked participants whether they had developed any gastrointestinal or neurological symptoms during the outbreak (i.e., November 4–16) and about any treatment they may have received for their illness. Participants who reported symptoms were asked to describe the ingredients of the last meal before they developed symptoms. Participants who were not ill were asked to describe what they ate during the 48 hours prior to the survey because we assumed their daily diet varied little before and after the outbreak. If participants had eaten seafood, we asked them to quantify the number of shellfish, fish, or shrimp they consumed. Bilingual staff used a questionnaire written in Spanish (the local language) to interview inhabitants. We also asked participants to describe where they obtained any seafood they ate during the relevant time period.

### PSP case-definition

We defined possible PSP case-patients as any Maderas Negras Island resident who developed at least one neurological symptom (i.e., paresthesia, circumoral numbness, dysarthria, dysphagia, ataxia, diplopia, dizziness, incoherent speech, light-headedness, and difficulty breathing) during November 4–16. We defined probable case-patients as those who met the possible PSP case-definition and had an elevated concentration of saxitoxin/g creatinine in their urine. We defined controls as a Maderas Negras Island resident who denied having any symptoms during November 4–16. We excluded infants aged < 1 year because we assumed they were primarily breast-feeding and may have been at lower risk of direct exposure to seafood.

### Laboratory analyses of shellfish, algae, and urine samples

To ascertain PSP toxin exposure, we tested shellfish, algae, and case-patient urine specimens for the presence of saxitoxins. We obtained shellfish samples from Maderas Negras Island harvesting sites implicated by case-patients as the source of their last meal before illness onset. We did not anticipate finding any meal remnants one week after the onset of the outbreak because few Maderas Negras Island residents own refrigerators. Each of the three shellfish samples collected contained ~20 clams, similar to the median serving size per person. Shellfish and algae were transported on dry ice to an FDA laboratory for analyses using the receptor binding assay [9] and high performance liquid chromatography [10], both of which are more specific tests than the mouse bioassay available in Nicaragua.

Blind-coded urine specimens were analyzed at the CDC using a commercially available enzyme-linked immunoassay for saxitoxin modified for the human urine matrix [11]. We tested 120 saxitoxin-negative anonymous urine specimens for both saxitoxin and creatinine to determine their upper 99% confidence limit. We defined a creatinine-corrected saxitoxin level that exceeded the upper 99% confidence limit (17.3 ng saxitoxin/g creatinine) as saxitoxin positive urine.

### Ethical considerations

The project was reviewed by CDC Human Subjects and was judged to be a public health investigation to identify the cause of the apparent poisonings and to assist the communities in developing plans to address the cause of these illnesses. Similarly, the activity was judged to be public health response rather than research by the Nicaraguan Health authorities.

### Consent

Informed consent was obtained from participants prior to administering the survey instrument.

## Results

### Demographics of Maderas Negras Island residents

During November 14–16, we surveyed 24 households with 107 residents aged ≥1 year. All agreed to participate in the survey. Half of the residents were female (53 [50%] of 107) and their median age was 16 years (interquartile range [IQR] 8–29 years). Of the 52 persons aged ≥18 years, 17 (33%) were homemakers, 13 (25%) were fishermen, nine (18%) were lumberjacks, seven (14%) were unemployed, and six (12%) had other occupations. Most (27 [52%]) of these 52 adults had partially completed primary school, 21 (40%) had no formal education, one (2%) had partially completed secondary school, one (2%) had completed secondary school, and one (2%) declined to provide information about their education.

### Case-patients

Ten (9%) of 107 participants developed at least one symptom during November 4–16 (Figure 2). All developed dizziness, nine (90%) developed numbness, 8 (80%) paresthesias, seven (70%) perioral numbness, six (60%) weakness, five (50%) difficulty breathing, five (50%) vomiting, five (50%) hypersalivation, four (40%) dysphagia, and three (30%) diarrhea. Nine (90%) met our case definition for possible PSP. Five (56%) of the nine possible PSP case-patients were hospitalized as a result of their illness and all survived.

### Risk factors

While all possible case-patients had eaten seafood, only 51 (52%) controls had eaten seafood 48 hours before the survey (OR = ∞, p = 0.005). While six (67%) of nine possible case-patients and 21 (21%) of 98 controls had eaten fish (p = 0.008), all case-patients and 17 (17%) of controls had eaten concha negra clams (*Anadara tuberculosa*) (P < 0.0001). There was no association between case status and other food or drink^a^ after accounting for concha negra intake. Some participants added lemon to their clams before eating them. Two of 13 (15%) people who cured the concha negra with lemon juice became ill whereas two of five (40%) people who did not cure the conchas became ill but this difference was not statistically significant (p = 0.1).

### Paralytic shellfish toxin laboratory analyses

The three shellfish samples obtained from Maderas Negras Island clam harvesting sites tested positive for saxitoxin by both testing methods. Toxicity in the clam samples reached up to 4,138 μg saxitoxin equivalents/100 g and saxitoxin was found to be the dominant toxin. Additionally, the two algal samples were positive for PSP toxicity according to the RBA and were found by HPLC to contain PSP toxins including saxitoxin, B1 (N-sulfocarbamoyl form of saxitoxin), and small amounts of neosaxitoxin. Study staff recovered one banked urine specimen collected 72 hours after a hospitalized case-patient developed numbness, paresthesias, and ataxia. The saxitoxin concentration in the hospitalized case-patient’s specimen urine was 20.8 ng saxitoxin/g creatinine and above the concentration of 17.3 ng saxitoxin/g creatinine considered to be indicative of exposure. The saxitoxin concentration in control patient’s specimen was 0.16 ng saxitoxin/g creatinine and below the assay’s cut-off of 17.3 ng saxitoxin/g creatinine.

## Discussion

Our findings suggest that residents of Maderas Negras Island were likely exposed to saxitoxins and developed PSP after eating concha negra clams during the November *P. bahamanse* harmful algal bloom. Case-patients with symptoms consistent with PSP were more likely to have eaten clams than controls and the one case-patient who provided a timely urine specimen demonstrated elevated saxitoxin urine concentrations when compared to control urine. This evidence suggests that Nicaraguan Ministry of Health’s prompt ban on shellfish consumption, harvesting, and export during the harmful algal bloom was warranted to prevent further intoxications.

Harmful algal blooms occur periodically in the coastal areas of Central America and are frequently associated with PSP outbreaks. In 1987, for example, an outbreak in Guatemala involving 187 cases and 26 deaths was reported after people consumed clam soup. The fatalities were the highest among young children (50%) compared with adults (7%) [12]. Given the episodic nature of harmful algal blooms, we recommend that authorities in regions where PSP is known to occur anticipate PSP cases during blooms and implement active surveillance for *P. bahamanse* in coastal waters*,* saxitoxins in local shellfish, and PSP among coastal residents to better determine when to temporarily ban shellfish harvesting and prevent intoxications. Components of such active surveillance would also be useful to determine when shellfish harvesting may safely resume after a harmful algal bloom dissipates.

The diagnosis of PSP has traditionally relied on clinical recognition of neurological symptoms and a history of shellfish ingestion because there was no human bioassay available to diagnose intoxication. In this investigation, the modified enzyme immunoassay employed detected an elevated level of saxitoxins in the urine of a suspected case-patient. Since the outbreak, more accurate and sensitive mass spectrometry assays have been developed [13]. Such assays may be valuable tools to rapidly determine the etiology of similar intoxications in the future.

Our study had several important limitations. Our ability to explore associations between illness and modifiable risk factors were limited by the small number of residents on Maderas Negras Island. Recall bias may have occurred as the investigative team arrived 12 days after the onset of the outbreak to obtain dietary histories from case-patients and some participants may have forgotten what they ate 48 hours before the onset of the outbreak. In addition, we did not believe that participants who were not ill would remember their diet before the outbreak and thus asked about their diet 48 hours before the survey when the shellfish harvesting ban was already in place. Furthermore, reporter bias may have been introduced as participants could have been reticent to inform the Ministry of Health team if they consumed concha negra after the November 6 shellfish ban. Nevertheless, island residents reported that they typically ate the same foods during their one or two daily meals regardless of the ban.

## Conclusions

Our findings suggest that during the harmful algal bloom of *P. bahamanse*, residents of Maderas Negras Island who ate concha negra clams were exposed to saxitoxins and developed PSP. Our findings also highlight the need for health authorities to institute active surveillance for Pyrodinium sp*.* in coastal waters*,* saxitoxins in local shellfish, and PSP among coastal residents during harmful algal blooms to quickly identify a PSP outbreak and prevent further PSP illnesses. Similar recommendations were made after intoxications in Central America during 1987 and 1989 when health officials proposed to establish a red tide surveillance system called *Red de vigilancia de las mareas rojas* (Hall S personal communication). Such surveillance and recent improvements in the detection of saxitoxins in urine may aid diagnosis, allowing health authorities to respond quickly to future outbreaks.

## Endnote

^a^rice, cheese, beans, tortillas, bread, bananas, shrimp, beef, pork, chicken, cream, or coffee.

## Abbreviations

PSP: Paralytic shellfish poisoning
IQR: Interquartile range
USD: United States dollars
CDC: Centers for Disease Control and Prevention
FDA: U.S. Food and Drug Administration
RBA: Receptor binding assay
HPLC: High performance liquid chromatography
OR: Odds ratio
